# Improved l-Leucine Production in *Corynebacterium glutamicum* by Optimizing the Aminotransferases

**DOI:** 10.3390/molecules23092102

**Published:** 2018-08-21

**Authors:** Li-Yan Feng, Jian-Zhong Xu, Wei-Guo Zhang

**Affiliations:** The Key Laboratory of Industrial Biotechnology, Ministry of Education, School of Biotechnology, Jiangnan University, 1800 Lihu Road, Wuxi 214122, China; feng_li_yan@163.com

**Keywords:** branched-chain amino acid aminotransferase, aspartate aminotransferase, *Corynebacterium glutamicum*, l-leucine

## Abstract

The production of branched-chain amino acids (BCAAs) is still challenging, therefore we rationally engineered *Corynebacterium glutamicum* FA-1 to increase the l-leucine production by optimizing the aminotransferases. Based on this, we investigated the effects of the native aminotransferases, i.e., branched-chain amino acid aminotransferase (BCAT; encoded by *ilvE*) and aspartate aminotransferase (AspB; encoded by *aspB*) on l-leucine production in C. glutamicum. The strain FA-1△*ilvE* still exhibited significant growth without leucine addition, while FA-1△*ilvE*△*aspB* couldn’t, which indicated that AspB also contributes to L-leucine synthesis in vivo and the yield of leucine reached 20.81 ± 0.02 g/L. It is the first time that AspB has been characterized for l-leucine synthesis activity. Subsequently, the aromatic aminotransferase TyrB and the putative aspartate aminotransferases, the *aspC*, *yhdR*, *ywfG* gene products, were cloned, expressed and characterized for leucine synthesis activity in FA-1△*ilvE*△*aspB*. Only TyrB was able to synthesize l-leucine and the l-leucine production was 18.55 ± 0.42 g/L. The two putative branched-chain aminotransferase genes, *ybgE* and *CaIlvE*, were also cloned and expressed. Both genes products function efficiently in BCAAs biosynthesis. This is the first report of a rational modification of aminotransferase activity that improves the l-leucine production through optimizing the aminotransferases.

## 1. Introduction

Branched-chain amino acids (BCAAs), i.e., l-leucine, l-valine and L-isoleucine, are three of eight essential amino acids [1] that cannot be synthesized in animals [2]. Recently, BCAAs have been used widely in medicine, food, and feed. Furthermore, BCAAs also play a vital role in human physiological functions and metabolism [3]. In industry, BCAAs are mainly produced by microbial fermentation employing mutant strains of bacteria, such as *Corynebacterium* sp. and *Escherichia* sp. [4]. Therefore, a BCAAs producer with excellent fermentability is needed for fermentation to increase the final titer and to reduce the production cost.

**Figure 1:**
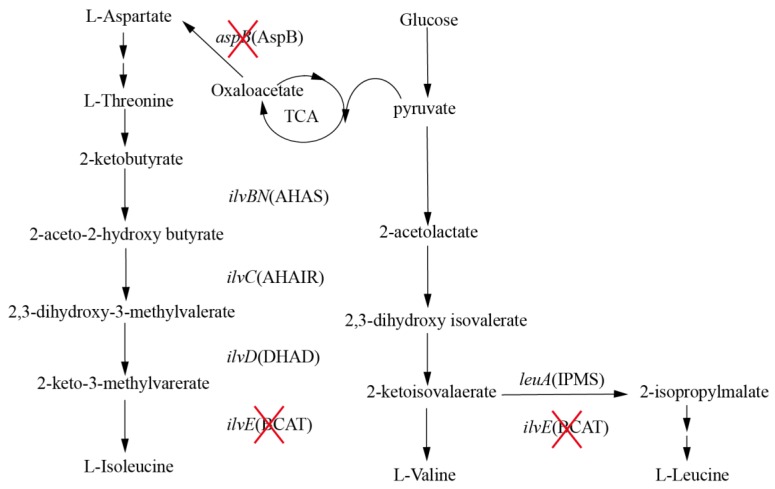
Biosynthesis of BCAAs. The genes and enzymes are shown in italic font and in parentheses. AHAS (*ilvBN*) acetohydroxyacid synthase, AHAIR (*ilvC*) acetohydroxyacid isomeroreductase, DHAD (*ilvD*) dihydroxyacid dehydratase, BCAT (*ilvE*) branched-chain amino acid aminotransferase, IPMS (*leuA*) 2-isopropylmalate synthase, AspB (*aspB*) aspartate aminotransferase. Deletion of genes and the respective proteins are indicated by “X”.

Unlike the biosynthetic pathways of other amino acids, the biosynthetic pathway of BCAAs includes branched and parallel reactions catalyzed by identical enzymes (Figure 1) [5]. l-Isoleucine and l-valine are synthesized from 2-ketobutyrate and pyruvate respectively, while l-leucine is branched from the 2-ketoisovalaerate in the valine pathway [6]. The synthesis of BCAAs comprises four reactions catalyzed by acetohydroxy acid synthase (AHAS; encoded by *ilvBN*), acetohydroxy acid isomeroreductase (AHAIR; encoded by *ilvC*), dihydroxyacid dehydratase (DHAD; encoded by *ilvD*) and branched-chain amino acid aminotransferase (BCAT; encoded by *ilvE*) [7,8]. In addition, the synthesis of l-leucine is still catalyzed by a series of enzymes in a branched pathway. Interestingly, the enzyme of final reaction pathway is BCAT, which synthesizes different BCAAs. It is obvious that production of one BCAA is often accompanied by accumulation of the other two BCAAs as the identical enzymes participate in the biosynthetic pathway of BCAAs, thereby reducing the final titer and increasing the difficulty of extraction [9].

Previous studies have shown that the final biosynthetic step of most amino acids involves the transamination reaction which is catalyzed by PLP-dependent aminotransferases [10]. The transamination reaction is reversible and consists of two half-reactions. Firstly, the amino group of substrates is transferred onto PLP to produce 5′-phosphate pyridoxamine (PMP) and keto acid (aldehyde). Subsequently, keto acid (aldehyde) accepts the amino group of PMP to produce the amino substrate and to regenerate PLP [11,12]. In 1950, Cammarata et al. [13] and Feldman et al. [14] first proposed the transamination of BCAAs in animals and microorganisms. However, the enzyme responsible for this reaction was not characterized until 1966 [15,16]. Ichihara et al. [15] and Taylor et al. [16] reported that the transamination of three BCAAs was catalyzed by BCAT. So far, the biosynthetic pathway of BCAAs has been characterized in *Escherichia coli*, *Salmonella typhimurium*, *Corynebacterium glutamicum*, *Bacillus subtilis* [17,18,19] and other strains. It was considered that the BCAT catalyzed the reactions between BCAAs and the respective keto-acids in these organisms [20]. Pyridoxal 5′-phosphate-dependent BCAT (EC 2.6.1.42, PLP dependent-BCAT) [21] encoded by *ilvE* catalyzes the transfer of the amino group from glutamic acid to keto acids in *Corynebacterium glutamicum.* PLP-dependent enzymes are divided into five types [22]. Aminotransferases occurred in the types I and IV, and BCAT belonged to PLP fold type IV [21,23].

Bacteria possess a number of different aminotransferases according to the KEGG and UniProtKB entries [20]. However, only some of the aminotransferases have been characterized because of the extensive and overlapping substrate specificities, which are involved in the synthesis of amino acids [24]. Moreover, the general description listed in the UniProtKB entries does not elucidate the definite functions of the enzymes. If one of aminotransferases is absent, it usually results in the nonexistence of a phenotype [20]. Based on the above-mentioned results, we here deleted the *ilvE* and *aspB* genes in l-leucine producer *C. glutamicum* FA-1 to construct a strain FA-1△*ilvE*△*aspB* with no l-leucine production, and different types of aminotransferases were overexpressed to search for the specific aminotransferases to improve the l-leucine biosynthesis. The results indicate that the presence of specific aminotransferase can improve the production of leucine.

## 2. Results

### 2.1. Effect of Inactivation of ilvE Gene on BCAAs

**Figure 2:**
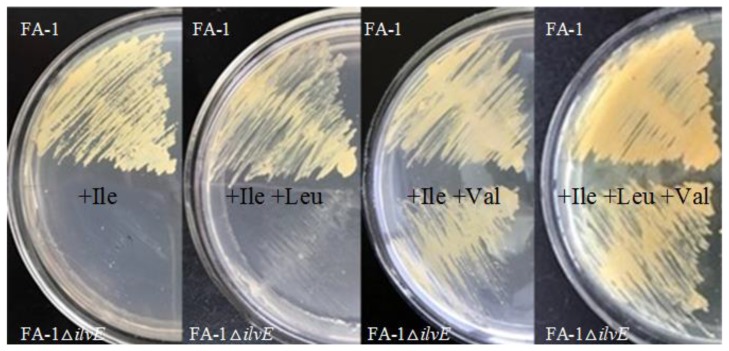
Growth of different *C. glutamicum* strains on different medium. At the top is shown the *C. glutamicum* FA-1, and the *C. glutamicum* FA-1△*ilvE* is shown at the bottom. Growth was carried out on medium CGXIIG containing l-methionine with amino acids supplemented as indicated (each at 0.1 g/L).

The strain *C. glutamicum* FA-1 is a high-producing strain, which is used to produce l-leucine. The strain FA-1 is auxotrophic for l-isoleucine, which reduces the effect of l-isoleucine on l-leucine production (see Section 4). To interrupt the accumulation of BCAAs, we eliminated the BCAT activity by deleting the *ilvE* gene in *C. glutamicum* FA-1. In order to analyze l-leucine and/or l-valine synthesis in *C. glutamicum* FA-1△*ilvE*, we assayed growth on CGXIIG (CGXII medium containing glucose) [25] medium supplemented with different BCAAs, respectively. As shown in Figure 2, *C. glutamicum* FA-1△*ilvE* grew normally in CGXIIG medium supplemented with isoleucine plus valine or three BCAAs. In contrast, *C. glutamicum* FA-1△*ilvE* showed no significant growth in CGXIIG medium with isoleucine and leucine. This result showed that the strain *C. glutamicum* FA-1△*ilvE* was fully dependent on l-valine supply but not on the supply of l-leucine, and strongly reduced in growth without valine.

**Figure 3:**
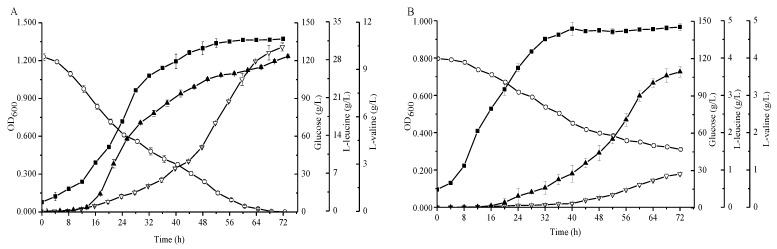
Comparison of the *C. glutamicum* strains FA-1 and FA-1△*ilvE* during cultivation in shake-flasks with fermentation medium. (**A**) *C. glutamicum* FA-1, (**B**) *C. glutamicum* FA-1△*ilvE*. Solid squares: OD_600_, Hollow circles: Glucose, Solid triangles: l-leucine, Hollow triangles: l-valine. The data represent mean values and standard deviations obtained from three independent cultivations.

The agar plate experiments demonstrated that an additional enzyme which has the ability to synthesize leucine must exist in strain FA-1△*ilvE*. Subsequently, we fermented the strains FA-1 and FA-1△*ilvE* in shake flasks to demonstrate their productivity. As shown in Figure 3, the l-leucine concentrations of *C. glutamicum* FA-1 and *C. glutamicum* FA-1△*ilvE* were 28.11 ± 0.29 and 3.63 ± 0.14 g/L, respectively. In addition, the l-valine concentration of *C. glutamicum* FA-1 was 10.43 ± 0.26 g/L, whereas the l-valine concentration of *C. glutamicum* FA-1△*ilvE* was less than 1 g/L. Moreover, the glucose decreased rapidly during the growth phase and was completely consumed within 72 h for *C. glutamicum* FA-1. In contrast, the consumption of glucose was at a low level for *C. glutamicum* FA-1△*ilvE*, and the by-products were markedly increased because the pathway of glucose to BCAAs was blocked (Figure 5D). Due to the deletion of *ilvE* gene, the recombinant strain showed decreased level of aminotransferase activity, but the enzyme data clearly showed that *C. glutamicum* FA-1△*ilvE* still had weak activities for the formation of l-leucine (Table 1).

Based on the abovementioned results, we found that the productivity of l-valine greatly decreased after deletion of *ilvE* gene and drastically less valine had been produced, which cannot maintain the growth of strain FA-1△*ilvE*. Therefore, the normal growth could be restored by adding additional l-valine and l-isoleucine to the medium. The remaining ability to produce the l-leucine suggested that there maybe are other aminotransferases acting on transamination to biosynthesize L-leucine in FA-1△*ilvE*.

### 2.2. Effect of aspB on l-Leucine Production

**Figure 4:**
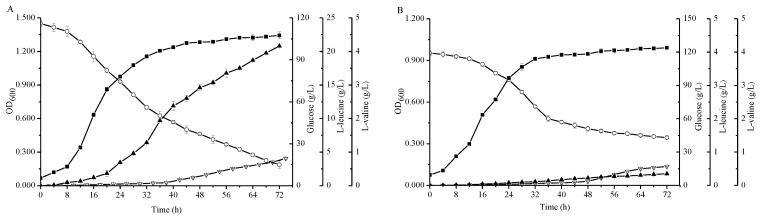
Comparison of the *C. glutamicum* strains FA-1△*ilvE*/pEC-XK99E-*aspB* and FA-1△*ilvE*△*aspB* during cultivation in shake-flasks with fermentation medium. (**A**) *C. glutamicum* FA-1△*ilvE*/pEC-XK99E-*aspB*, (**B**) *C. glutamicum* FA-1△*ilvE*△*aspB*. Solid squares: OD_600_, Hollow circles: Glucose, Solid triangles: l-leucine, Hollow triangles: l-valine. The data represent mean values and standard deviations obtained from three independent cultivations.

**Figure 5:**
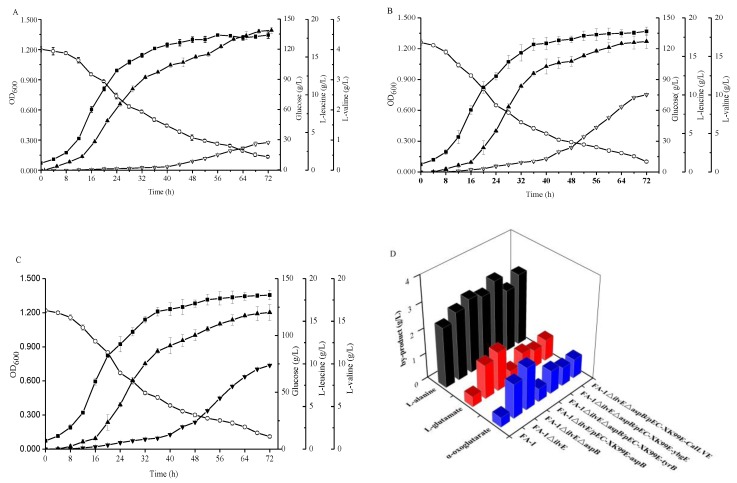
Comparison of the different strains during cultivation in shake-flasks with fermentation medium. (**A**) *C. glutamicum* FA-1△*ilvE*△*aspB*/pEC-XK99E-*tyrB*, (**B**) *C. glutamicum* FA-1△*ilvE*△*aspB*/pEC-XK99E-*ybgE*, (**C**) *C. glutamicum* FA-1△*ilvE*△*aspB*/pEC-XK99E-*CaIlvE*, (**D**) by-products. Solid squares: OD_600_, Hollow circles: Glucose, Solid triangles: l-leucine, Hollow triangles: l-valine. The data represent mean values and standard deviations obtained from three independent cultivations.

Due to the fact *C. glutamicum* FA-1△*ilvE* still had weak activities for the formation of L-leucine, we studied the effects of many native aminotransferase genes from *C. glutamicum* FA-1 on BCAAs (data not shown). Among them, an aspartate aminotransferase (AspB) -coding gene *aspB* was cloned and expressed in *ilvE* strain to construct the recombinant strain *C. glutamicum* FA-1△*ilvE*/pEC-XK99E-*aspB*. Compared with the strain *C. glutamicum* FA-1△*ilvE*, the leucine production was enhanced to 20.81 ± 0.02 g/L by *C. glutamicum* FA-1△*ilvE*/pEC-XK99E-*aspB* (Figure 4). Interestingly, there was little change in the l-valine production as compared with *C. glutamicum* FA-1△*ilvE*. Moreover, the recombinant strain *C. glutamicum* FA-1△*ilvE*/pEC-XK99E-*aspB* exhibited much higher activity with leucine than the *ilvE*^-^ strain (Table 1). On the other hand, the consumption of glucose was significantly higher than that of *C. glutamicum* FA-1△*ilvE*, whereas the by-products declined (Figure 5D). These results demonstrated that the aminotransferase-coding gene *aspB* could catalyze the biosynthesis of l-leucine. This may be why the strain *C. glutamicum* FA-1△*ilvE* could grow in the CGXIIG without exogenous l-leucine. To further determine the effect of *aspB* on l-leucine production, we inactivated the *aspB* gene based on the *ilvE*^-^ mutant strain to construct a double mutant *C. glutamicum* FA-1△*ilvE*△*aspB*, and assayed growth performance on CGXIIG with different three BCAAs and aspartate. *C. glutamicum* FA-1△*ilvE*△*aspB* was fully dependent on the supply of three BCAAs plus aspartate (data not shown). In addition, activities in crude extracts of strains FA-1△*ilvE* and FA-1△*ilvE*△*aspB* grown on the CGXIIG were compared (Table 1), indicating that there is no detectable aminotransferase activity in strain FA-1△*ilvE*△*aspB*. Moreover, the glucose consumption of *C. glutamicum* FA-1△*ilvE*△*aspB* was lowest, and there were hardly any BCAAs during the fermentation (Figure 4).

By comparing the fermentation performance of *C. glutamicum* FA-1△*ilvE*/pEC-XK99E-*aspB*, *C. glutamicum* FA-1△*ilvE*, we found that the production of leucine was increased by expressing the gene *aspB*. Based on this, we speculated that AspB is also involved with l-leucine synthesis activity except for BCAT. From the Table 1, we found that BCAT was the important enzyme for l-leucine formation in parent strain, while the AspB exhibited lower enzyme activity. In addition, we performed growth experiments with strain FA-1△*ilvE*△*aspB*, the strain FA-1△*ilvE* still exhibited significant growth without leucine addition (Figure 1), while FA-1△*ilvE*△*aspB* couldn’t (data not shown), indicating that the growth of *ilvE*^-^ strain did not require leucine supply owing to the presence of *aspB*, thus confirming that this inference was correct.

### 2.3. Effect of Different Aminotransferases on the Biosynthesis of l-Leucine or l-Valine

According to literature and database display, *E. coli*-derived aminotransferases gene *tyrB* and *aspC* encode aromatic amino acid aminotransferase and aspartate aminotransferase [26,27], respectively. *B. subtilis*-derived aminotransferase genes *ywfG* [28] and *yhdR* encode putative aspartate amino aminotransferases, and *ybgE* gene encodes BCAT [28]. The aminotransferase gene *CaIlvE* in *C. acetobutylicum* may encode the BCAT. The reason for interest in these aminotransferases is that due to the broad and overlapping substrate specificities, an aminotransferase could be involved in the synthesis of many amino acids. However, the affinity for substrates was different, which could lead to catalysis of different reactions in a particular environment by the same enzyme [29,30]. To search the specific aminotransferases for l-leucine or l-valine biosynthesis, the PLP-dependent fold type I aminotransferases TyrB, AspC, YwfG, and YhdR, and fold type IV enzymes YbgE and *Ca*IlvE were investigated in the *C. glutamicum* FA-1△*ilvE*△*aspB* strain. The recombinant strains were cultured in shake flask fermentation containing three branched-chain amino acids and aspartate, respectively. As shown in Figure 5, the maximum l-valine production by *C. glutamicum* FA-1△*ilvE*△*aspB*/pEC-XK99E-*ybgE* and *C. glutamicum* FA-1△*ilvE*△*aspB*/pEC-XK99E-*CaIlvE* reached 10.04 ± 0.06 and 9.83 ± 0.11 g/L, respectively, indicating that the production of l-valine can be enhanced by overexpression of *ybgE* and *CaIlvE* genes. However, there was no significant increase in l-valine production by other recombinant strains as compared with *C. glutamicum* FA-1△*ilvE*△*aspB* (Table 2).

With the overexpression of these genes, the l-leucine production by *C. glutamicum* FA-1△*ilvE*△*aspB*/pEC-XK99E-*tyrB*, *C. glutamicum* FA-1△*ilvE*△*aspB*/pEC-XK99E-*ybgE* and *C. glutamicum* FA-1△*ilvE*△*aspB*/pEC-XK99E-*CaIlvE* was increased to 18.55 ± 0.42, 16.94 ± 0.29 and 16.03 ± 0.92 g/L, respectively (Figure 5). However, there were no differences in l-leucine production between other recombinant strains and *C. glutamicum* FA-1△*ilvE*△*aspB* (Table 2). In Table 2, we observe that the parameters of l-leucine and l-valine showed no obvious differences in strains FA-1△*ilvE*△*aspB*/pEC-XK99E-*aspC*, FA-1△*ilvE*△*aspB*/pEC-XK99E-*yhdR* and FA-1△*ilvE*△*aspB*/pEC-XK99E-*ywfG*. In addition, as shown in Table 3, the specific activities of aminotransferases in only three recombinant strains were higher than that of *C. glutamicum* FA-1△*ilvE*△*aspB*. This showed that the activity of aminotransferases was consistent with the production of amino acids. Figure 5D shows the by-products accumulation, indicating that the concentration of by-products was similar in the three recombinant strains FA-1△*ilvE*△*aspB*/pEC-XK99E-*tyrB*, FA-1△*ilvE*△*aspB*/pEC-XK99E-*ybgE* and FA-1△*ilvE*△*aspB*/pEC-XK99E-*CaIlvE*.

In a word, the results indicated that the product of *tyrB* gene exhibited high specificity for the biosynthesis of l-leucine but no activity for l-valine. On the other hand, the aminotransferases encoded by *ybgE* and *CaIlvE* genes can catalyze the transamination reactions between the l-leucine as well as l-valine and keto acid. However, the *aspC*, *yhdR* and *ywfG* encoding aminotransferases cannot catalyze the synthesis of l-leucine and l-valine.

## 3. Discussion

Aminotransferases catalyze the transfer of the amino group from glutamate to the precursors of the target amino acids in bacteria*.* BCAT, usually considered the last enzyme in BCAA sythesis, plays a vital role in the biosynthesis of BCAAs. In this study, we found that the BCAT-deficient strain *C. glutamicum* FA-1△*ilvE* exhibited the same cell growth rate during cultivating in CGXIIG media with or without l-leucine except for with l-isoleucine and l-valine, and accumulated a certain amount of l-leucine in fermentation broth (Figure 2). These results indicated that other aminotransferases must be involved in catalyzing the synthesis of l-leucine in vivo. After further investigation, we speculated that AspB is also involved in l-leucine synthesis in addition to BCAT, although BCAT was the major enzyme for l-leucine formation in parent strain, while the AspB exhibited lower enzyme activity.

Son et al. [31] have reported the crystal structural of the AspB (PDB code:5IWQ) from *C. glutamicum* ATCC 13032, and found that it is a homodimer protein, and the monomer consists of the core domain and the auxiliary domain. However, functional studies on this enzyme have not been reported on so far. For the first time we provided convincing proof that AspB was characterized for l-leucine synthesis activity. The production of l-leucine between FA-1△*ilvE* and the recombinant FA-1△*ilvE*/pEC-XK99E-*aspB* strains (Figure 3 and Figure 4) indicated that the AspB contributed to l-leucine synthesis. In addition, the *ilvE*^-^ *and aspB*^-^ double mutant strain required three branched-chain amino acids to maintain the cell growth (data not shown) and the l-leucine production of double mutant was less than 1 g/L (Figure 4), which strongly indicated that it is because of the presence of the AspB that the strain FA-1△*ilvE* can be grown in minimal medium without l-leucine.

In *E. coli*, the structure and functions of AspC and TyrB had been characterized [10,26,32]. In this study, we also studied the effect of AspC and TyrB on BCAAs production in *C. glutamicum*, and the results showed that TyrB had the specificity for l-leucine production, whereas AspC exhibited no activity on BCAA production (Table 4). This result was consistent with the study in *E. coli* [26]. Although both AspC and AspB belong to class I of aspartate aminotransferases, AspB is further sub-classified to subgroup Ic rather than subgroup Ia and subgroup Ib based on the special structure and the lower similarity of amino acid sequence with the other AspATs (aspartate aminotransferases) [31]. Compared with the other AspATs, AspB exhibited unique residues to stabilize the substrate and PLP cofactors [31]. The AspB may belong to the MocR subfamily of GntR-type helix-turn-helix transcriptional regulators according to the Pfam database. Based on this, the research on AspB still must be carried out. Moreover, YbgE and CaIlvE have the ability to synthesize the l-leucine and l-valine (Figure 5), and they both belong to the branched-chain amino acid family. For the current study on the BCAT, the existing BCATs act on three BCAAs in microorganisms [20,28,33,34,35], but whether the class enzymes can catalyze the synthesis of BCAAs still require further study due to the broad substrate specificities [15]. However, the YwfG and YhdR did not exhibit activity in the synthesis of l-leucine and l-valine.

Under the experimental conditions, we fermented all strains in shake flasks to demonstrate their productivity. With the inactivation of *ilvE* gene, the concentration of l-valine was less than 1 g/L by *C. glutamicum* FA-1△*ilvE*, and l-leucine production was reduced by 87.09% (from 28.11 to 3.63 g/L). These results indicate that the strain FA-1△*ilvE* still has another enzyme which can synthesize l-leucine. With *aspB* overexpressed, the l-leucine production was increased by 82.56% (from 3.63 to 20.81 g/L) by *C. glutamicum* FA-1△*ilvE*/pEC-XK99E-*aspB*, and the l-valine production was not changed. With *aspB* inactivated, the double mutant was auxotrophic for three BCAAs. Moreover, l-leucine and valine production were both less than 1 g/L by *C. glutamicum* FA-1△*ilvE*△*aspB*. These results indicating that AspB functions in synthesis of l-leucine and had no activities on l-valine. In conclusion, we developed the high-yielding strain to improve l-leucine and demonstrated the importance of aminotransferases involved in transamination reaction for the BCAAs production. Moreover, the present work firstly points out that AspB is also involved in l-leucine synthesis except for BCAT. In addition, we also found that the TyrB not only participates in the biosynthesis of l-leucine in *E. coli*, but also plays the same role in *C. glutamicum*. However, the YbgE and CaIlvE enzymes also have activity toward l-leucine and l-valine production, while the aminotransferases AspC, YhdR and YwfG had no catalytic activity for BCAAs production in this study. The l-leucine production was increased to 18.55 ± 0.42 g/L by the recombinant strain FA-1△*ilvE*△*aspB*/pEC-XK99E-*tyrB*. 

These results indicated that optimizing the aminotransferases to switch its substrates specificities has great potential to improve the production of BCAAs, including leucine. However, the recombinant strain FA-1△*ilvE*△*aspB*/pEC-XK99E-*tyrB* accumulated a fair number of by-products with the low l-leucine productivity. Therefore, further optimizing l-leucine production with strain FA-1△*ilvE*△*aspB*/pEC-XK99E-*tyrB* will aim at increasing the carbon flux in l-leucine biosynthetic pathway by overexpressing the key enzymes in this pathway and/or at disrupting the biosynthetic pathway of by-products by deleting the key enzymes in by-products biosynthetic pathway. These results reported here could serve as a general concept and guidance for breeding high-yielding strains and producing l-leucine in industry.

## 4. Material and Methods

### 4.1. Bacterial Strains, Plasmids, and Growth Conditions

The bacteria and plasmids used in this study are listed in Table 4. The l-leucine-producing strain *C. glutamicum* FA-1 was derived from the wild-type strain *C. glutamicum* ATCC 13032, which was mutagenized by atmospheric and room temperature plasma (ARTP) biological breeding system (Si Qing Yuan Biotechnology Co., Ltd., Beijing, China). This strain was resistant to α-aminobutyric acid and α- thiazolylalanine, and auxotrophic for l-methionine and L-isoleucine. The *E. coli* strains grew in Luria-Broth (LB) medium at 37 °C with agitation at 100 rpm. LBG (LB supplemented with 5 g/L glucose) was used for *C. glutamicum* at 30 °C with agitation at 100 rpm. The minimal medium usually used for *C. glutamicum* was CGXII with 4% (*w*/*v*) glucose [25]. When appropriate, kanamycin (Kan 25 or 50 mg/L) was added to the medium. IPTG was added to a final concentration of 1 mmol/L.

For shake flask cultivation, the cells were grown in a 500 mL conical flask containing 50 mL seed medium for 12–18 h. The seed medium contained 30 g/L glucose, 25 g/L corn syrup, 5 g/L (NH_4_)_2_SO_4_, 1.3 g/L KH_2_PO_4_·3H_2_O, 0.4 g/L MgSO_4_·7H_2_O, 0.01 g/L MnSO_4_·H_2_O, 0.4 g/L methionine, 300 μg/L VB1, 200 μg/L VH, 10 g/L sodium citrate, 2 g/L urea, 10 g/L yeast extract and 20 g/L CaCO_3_. The fermentation medium contained 130 g/L glucose, 25 g/L corn syrup, 15 g/L (NH_4_)_2_SO_4_, 15 g/L CH_3_COONH_4_, 1.3 g/L KH_2_PO_4_·3H_2_O, 0.5 g/L MgSO_4_·7H_2_O, 0.01 g/L MnSO_4_·H_2_O, 0.06 g/L isoleucine, 0.7 g/L methionine, 0.5 g/L glutamic acid, 160 μg/L VB1, 50 μg/L VH, 2 g/L sodium citrate, 2 g/L urea and 30 g/L CaCO_3_. The fermentation medium was supplemented with 0.6 g/L valine to culture the *ilvE*^-^ strain. The *ilvE*^-^ *aspB*^-^ strain and recombinant strains were also grown in fermentation medium supplemented 0.6 g/L l-valine, 0.6 g/L l-leucine and 1 g/L l-aspartate. All cells were cultured at 30 °C and shaken at 100 rpm. The fermentation lasted for 72 h.

### 4.2. Construction of Plasmids

The aminotransferase genes *tyrB* and *aspC* were derived from *E. coli*, *aspB* gene was derived from *C. glutamicum*, *ybgE*, *yhdR* and *ywfG* genes were derived from *B. subtilis*, and *CaIlvE* was derived from *C. acetobutylicum*. The genes and homologous-arm fragments for gene deletion were amplified using the corresponding primers listed in Table 5. The up- and down-stream homologous fragments of *ilvE* were cloned into pK18mob*sacB* via its attached *Smal* I and *Sal* I sites, the up- and down-stream homologous fragments of *aspB* were cloned into pK18mob*sacB* via its attached *Eco*R I/*Hind* III sites. The plasmids harboring different aminotransferase genes were constructed using the method of Sambrook et al. [36]. The *tyrB* and *aspC* fragments were amplified using the *E. coli* W3110 as templates and the *aspB* (Gene ID: 1021066) fragment from *C. glutamicum* ATCC 13032 was amplified*.* The *ybgE*, *yhdR* and *ywfG* fragments and the *CaIlvE* fragment were amplified using *B. subtilis* 168 and *C. acetobutylicum* ATCC 824 as templates*.*

### 4.3. Construction of Strains

*C. glutamicum* harboring recombinant plasmids were constructed based on the method of van der Rest et al. [37]. The *ilvE*^-^ and *ilvE*^-^ *aspB*^-^ strains were made by using pK18mob*sacB*△*ilvE* and pK18mob*sacB*△*aspB*, respectively. Colonies were selected for Kan resistance to establish integration of the plasmid in the chromosome [38]. In the second round of positive selection by using sucrose resistance, colonies were selected for deletion of the vector [38]. The chromosome deletions were verified by PCR analysis using the primers according to the description of Table 5. The strategy used for allelic exchange in *C. glutamicum* was based on the method of Xu et al. [39].

### 4.4. Preparation of Cell Extracts and Enzyme Assays

Cell crude extracts were prepared as follows. Cell were harvested by centrifugation (12,000 rpm, 20 min) at 4 °C. *C. glutamicum* cells were suspended in 50 mM Tris-HCl, pH 8.0, and then cells were treated with lysozyme (20 mg/mL) at 37 °C for 1 h. Thus, the cell suspension was sonicated for 15 min on ice and the resultant sonicates were centrifuged at 12,000 rpm for 20 min at 4 °C, the supernatant fluid constituted the crude enzyme solution. The AT assay was based on the method of Marienhagen et al. [20]. Reaction mixtures contained, in a total volume of 1.0 mL, 100 mM Tris-HCl (pH 8.0), 0.25 mM pyridoxal-5′-phosphate, 5 mM α-ketoisovalerate or α-ketoisocaproate, and 10 mM sodium glutamate. The reaction was started by the addition of crude extract and was performed at 30 °C with 20 min. 30 μL of 5% perchloric acid was added to stop the reaction. The samples were centrifuged by the addition of 20 mM K_2_CO_3_ to remove the proteins and salts (12,000 rpm, 10 min, 4 °C). Subsequently, the l-leucine and l-valine were quantified by high-pressure liquid chromatography (HPLC). One enzyme unit was defined as that the amount of enzymes which converted 1 μmol l-leucine or l-valine per min at temperature of the assay. Protein concentrations were determined by the method of Lowry et al. [40].

### 4.5. Analytical Methods

Two milliliters of samples were taken from Erlenmeyer flasks every 4 h. One milliliter of samples was used to determine the biomass concentration by measuring the OD_600_ after an appropriate dilution or dry cell weight (DCW) per liter as described previously [41]. The other 100 μL of sample was diluted 100-fold to determine the glucose by an SBA-40E immobilized enzyme biosensor (Biology Institute of Shandong Academy of Sciences, Shandong, China). In addition, the supernatant of fermentation broth was also used to determine the concentration of amino acids and/or organic acids. The amino acids’ concentrations were determined by reversed-phase high-pressure liquid chromatography on an Agilent 1200 system (Agilent Technologies, Santa Clara, CA, USA) with DAD detection (338 nm) after automatic precolumn derivatization with *ortho*-phthaldialdehyde [41,42]. Separation was carried out at 40 °C on a dC18 column (particle size 5 μm, 4.6 mm × 250 mm, Thermo Fisher Scientific, Waltham, MA, USA). The elution buffer consisted of a polar phase (0.1 M sodium acetate, pH 7.2) and a nonpolar phase was prepared according to the description of Hou et al. [41]. Quantification was done by calculation of concentration using an internal standard. The organic acids’ concentrations were determined by HPLC (Agilent 1200) with UV detection (215 nm). Separation was carried out at 25 °C on a dC18 column (particle size 5 μm, 4.6 mm × 250 mm, Waters, Milford, MA, USA). The elution buffer consisted of a polar phase (0.01 moL/L KH_2_PO_4_) and a nonpolar phase (5% acetonitrile). Quantification was done by calculation of the peak area by the external standard method [41].

## Figures and Tables

**Table 1:** Specific activities of transamination enzymes with leucine and valine as substrates.

Strain	Growth Conditions ^a^	Aminotransferase Specific Activity (mU/mg of Protein)	
		Leucine	Valine
FA-1	+Ile	18.12 ± 2.12	10.07 ± 1.87
FA-1△*ilvE*	+Ile + Val	2.73 ± 0.92	<1
FA-1△*ilvE*△*aspB*	+Ile + Val + Leu + Asp	<1	<1
FA-1△*ilvE*/pEC-XK99E-*aspB*	+Ile + Val	17.39 ± 2.67	<1

^a^ The medium CGXIIG contained l-methionine. All data represent values of three determinations of three independent experiments ± SD.

**Table 2:** Comparisons of shake flask culture parameters of BCAAs production by different strains.

Strain	l-Leucine (g/L)	l-Valine (g/L)	l-Alanine (g/L)
FA-1△*ilvE*△*aspB*	<1	<1	2.75 ± 0.23
FA-1△*ilvE*△*aspB/*pEC-XK99E-*aspC*	<1	<1	2.98 ± 0.72
FA-1△*ilvE*△*aspB/*pEC-XK99E-*yhdR*	<1	<1	2.69 ± 0.11
FA-1△*ilvE*△*aspB/*pEC-XK99E-*ywfG*	<1	<1	2.45 ± 0.35

All data represent values of three determinations of three independent experiments with ±SD.

**Table 3:** Specific activities of transamination enzymes with leucine and valine as substrates.

Strain	Growth Conditions ^a^	Aminotransferase Specific Activity (mU/mg of Protein)	
		Leucine	Valine
FA-1△*ilvE*△*aspB*/pEC-XK99E-*tyrB*	+Ile + Val + Leu + Asp	16.85 ± 1.19	<1
FA-1△*ilvE*△*aspB*/pEC-XK99E-*ybgE*	+Ile + Val + Leu + Asp	16.11 ± 2.01	8.79 ± 1.33
FA-1△*ilvE*△*aspB*/pEC-XK99E-*CaIlvE*	+Ile + Val + Leu + Asp	15.94 ± 1.88	8.33 ± 1.28
FA-1△*ilvE*△*aspB*/pEC-XK99E-*aspC*	+Ile + Val + Leu + Asp	<1	<1
FA-1△*ilvE*△*aspB*/pEC-XK99E-*yhdR*	+Ile + Val + Leu + Asp	<1	<1
FA-1△*ilvE*△*aspB*/pEC-XK99E-*ywfG*	+Ile +Val + Leu + Asp	<1	<1

^a^ The medium CGXIIG contained l-methionine. All data represent values of three determinations of three independent experiments with ± SD.

**Table 4:** Strains and plasmids used.

Strains and Plasmid	Description	Source or Reference
Strains		
*E. coli*		
BL21(DE3)	F^-^ ompT gal dcm lon hsdS_B_ (r_B_^-^m_B_^-^) λ(DE3)	Strata gene
W3110	Wild type	Lab stock
*C. glutamicum*		
ATCC 13032	Type strain	ATCC
FA-1	*ilvE* ^+^ *aspB* ^+^	Lab stock
FA-1△*ilvE*	As in FA-1, *ilvE*^-^	This work
FA-1△*ilvE*/pEC-XK99E-*aspB*	As in FA-1△*ilvE*, *aspB*^+^	This work
FA-1△*ilvE*△*aspB*	As in FA-1△*ilvE*, *aspB*^-^	This work
FA-1△*ilvE*△*aspB*/pEC-XK99E-*tyrB*	As in FA-1△*ilvE*△*aspB*, *tyrB*^+^	This work
FA-1△*ilvE*△*aspB*/pEC-XK99E*-ybgE*	As in FA-1△*ilvE*△*aspB*, *ybgE*^+^	This work
FA-1△*ilvE*△*aspB*/pEC-XK99E*-CaIlvE*	As in FA-1△*ilvE*△*aspB*, *CaIlv* ^+^	This work
FA-1△*ilvE*△*aspB*/pEC-XK99E-*aspC*	As in FA-1△*ilvE*△*aspB*, *aspC*^+^	This work
FA-1△*ilvE*△*aspB*/pEC-XK99E-*yhdR*	As in FA-1△*ilvE*△*aspB*, *yhdR*^+^	This work
FA-1△*ilvE*△*aspB*/pEC-XK99E-*ywfG*	As in FA-1△*ilvE*△*aspB*, *ywfG* ^+^	This work
*B. subtilis* 168	Wild type	ATCC
*C. acetobutylicum* ATCC 824	Wild type	ATCC
Plasmids		
pk18mob*sacB*	Integration vector	Lab stock
pk18mob*sacB*-△*ilvE*	pk18mob*sacB* carrying *ilvE*-L and *ilvE*-R gene	This work
pk18mob*sacB*-△*aspB*	pk18mob*sacB* carrying *aspB* -L and *aspB* -R gene	This work
pEC-XK99E	*E. coli*-*C. glutamicum* shuttle vector and Kan^r^	Lab stock
pEC-XK99E-*aspB*	pEC-XK99E with a 1.3 kb *Kpn* I/*Xba* I fragment containing *aspB* gene	This work
pEC-XK99E-*tyrB*	pEC-XK99E with a 1.2 kb *Eco*R I/*Bam*H I fragment containing *tyrB* gene	This work
pEC-XK99E*-ybgE*	pEC-XK99E with a 1.0 kb *Eco*R I/*Bam*H I fragment containing *ybgE* gene	This work
pEC-XK99E*-CaIlvE*	pEC-XK99E with a 1.0 kb *Eco*R I/*Bam*H I fragment containing *CaIlvE* gene	This work
pEC-XK99E*-aspC*	pEC-XK99E with a 1.2 kb *Eco*R I/*Bam*H I fragment containing *aspC* gene	This work
pEC-XK99E*-yhdR*	pEC-XK99E with a 1.1 kb *Eco*R I/*Bam*H I fragment containing *yhdR* gene	This work
pEC-XK99E*-ywfG*	pEC-XK99E with a 1.2 kb *Kpn* I/*Xba* I fragment containing *ywfG* gene	This work

**Table 5:** Primers used in this work.

Primer	Sequence (5′ → 3′)	Description or Reference
P1	TCCCCCGGGCAAGCCTAGCCATTCCTC (*Smal* I)	P1 to P4, primers for *ilvE* deletion
P2	GCTCTAGACGTCTACCAGCAGTTCAAG (*Xba* I)	
P3	GCTCTAGATGGGATACGAAGTAGAAGAGC (*Xba* I)	
P4	ACGCGTCGACTTTCCAACCGTCAGCTG (*Sal* I)	
P5	ATGACGTCATTAGAGTTCA	P5 and P6: primers for *ilvE* deletion identification
P6	GGTCTTAAAACCGGTTGAT	
P7	GGGGTACCATGAGTTCAGTTTCGCTGC (*Kpn* I)	P7 and P8: primers for *aspB* and *aspB* deletion identification
P8	GCTCTAGATCTCCGCTGTATTCACTTTTAG (*Xba* I)	
P9	GGAATTCTATCTTGTGAACTCCCCCAG (*Eco*R I)	P9 to P12, primers for *aspB* deletion
P10	ACGCGTCGACTATCAACGATGCCATCCAG (*Sal* I)	
P11	ACGCGTCGACCCGAAGTTCAACAAGGTTCTG (*Sal* I)	
P12	CCCAAGCTTGGCCAGGCTCAAAATCTC (*Hind* III)	
P13	GGAATTCTGGAGAACCATCGCATGTTTC (*Eco*R I)	P13 and P14: primers for *tyrB*
P14	CGGGATCCTAATTTCACTGCAGGCTGGG (*Bam*H I)	
P15	GGAATTCATGAATAAGCTTATTGAACGAG (*Eco*R I)	P15 and P16: primers for *ybgE*
P16	CGGGATCCTCACACTTCCACTGTCCAG (*Bam*H I)	
P17	GGAATTCCAGCGTTAATCTACTCATCATG (*Eco*R I)	P17 and P18: primers for *CaIlvE*
P18	CGGGATCCTTTGCAACAGCCCATTC (*Bam*H I)	
P19	GGAATTCATGAATAAGCTTATTGAACGAG (*Eco*R I)	P19 and P20: primers for *aspC*
P20	CGGGATCCTTACAGCACTGCCACAATCG (*Bam*H I)	
P21	GGAATTCATGAAATTGGCTGGGTTATC (*Eco*R I)	P21 and P22: primers for *yhdR*
P22	CGGGATCCTGGATTGGAAGAGGAAGG (*Bam*H I)	
P23	GGGGTACCATGGAAATAACACCGTCC (*Kpn* I)	P23 and P24: primers for *ywfG*
P24	GCTCTAGATTAGCGGGATGTTTCTTG (*Xba* I)	

Underlining shows the restriction site for the enzyme indicated in parentheses.
